# Severe complications in the induction phase of therapy in a pediatric patient with T-cell acute lymphoblastic leukemia: A case report

**DOI:** 10.1097/MD.0000000000034965

**Published:** 2023-09-08

**Authors:** Agata Rocka, Magdalena Woźniak, Monika Lejman, Joanna Zawitkowska

**Affiliations:** a Pediatric Radiology, Medical University of Lublin, Lublin, Poland; b Laboratory of Genetic Diagnostics, Medical University of Lublin, Lublin, Poland; c Department of Paediatric Haematology, Oncology, and Transplantology, Medical University, Lublin, Poland.

**Keywords:** acute lymphoblastic leukemia, chemotherapy toxicity, cholecystitis, pediatrics

## Abstract

**Rationale:**

Acute lymphoblastic leukemia (ALL) represents approximately 1-quarter of all new cases of childhood cancer. Although overall survival following diagnosis has improved in recent years, the toxicity of chemotherapy remains a concern.

**Patient concerns:**

We describe an 11-year-old male patient diagnosed with T-cell precursor ALL who developed compounded complications during the induction phase of chemotherapy. Patient was hospitalized in the Department of Pediatric Hematology, Oncology, and Transplantology of the Medical University of Lublin, Poland. The patient’s induction therapy was started according to the AIEOP-BFM ALL 2017 protocol IAp (International Collaborative Treatment Protocol for Children and Adolescents with Acute Lymphoblastic Leukemia).

**Diagnoses:**

Patient developed compounded complications such as cholecystitis, hepatotoxicity, pancreatitis and myelosuppression.

**Interventions:**

The patient was treated with leukapheresis, received a broad-spectrum antibiotic, potassium supplementation and hepatoprotective treatment and laparotomy cholecystectomy.

**Outcomes:**

In the available literature, there is a limited amount of similar clinical cases with multiple complications in pediatric patients with ALL. Toxicities cause delays in the treatment of the underlying disease.

**Lessons:**

In children with acute lymphoblastic leukemia, there are side effects during the treatment such as cholecystitis and pancreatitis. Complications during treatment require a quick response and modification of disease management. Abdominal ultrasound performed before treatment makes it possible to observe the dynamics of lesions. Genetic mutation analysis could allow us to more precisely respond to the possible susceptibility to and appearance of complications after the use of a given chemotherapeutic agent.

## 1. Introduction

Acute lymphoblastic leukemia (ALL) represents 24.9% of all new cases of childhood cancer, diagnosed most frequently in children from 1 to 4 years old.^[1]^ In the last thirty years, there has been significant improvement in the treatment of pediatric acute lymphoblastic leukemia. The proportion of patients surviving for 5 years exceeds 90% in many developed countries.^[2]^ Acute T-cell lymphoblastic leukemia (T-ALL) accounts for approximately 10% to 15% of diagnosed cases of ALL in pediatric patients.^[3]^ Despite the improvement in overall survival following diagnosis, the toxicity toxicities of chemotherapy remains a concern. The risk of death from toxic effects during chemotherapy is 3.2%.^[4]^ The 5-year overall survival for acute lymphoblastic leukemia in pediatrics after undergoing treatment is 81.6% for BCP-ALL and 71.4% for T-ALL.^[5]^ The overall relapse rate is 11.20%.^[6]^ We report a child with T-ALL who developed cholecystitis, hepatitis and pancreatitis during chemotherapy. Medical records of this patient were reviewed for clinical and laboratory data. The patient was hospitalized in the Department of Pediatric Hematology, Oncology and Transplantology of the Medical University of Lublin, Poland.

**Figure 1.**
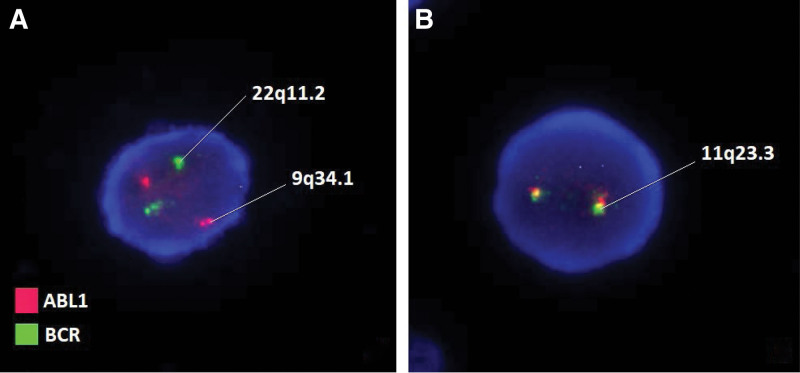
Fluorescence in situ hybridization (FISH) probe of the described patient’s case. (A) Mo-lecular Abbott. Vysis LSI BCR/ABL dual color, dual fusion translocation probe hybridized to a nucleus containing a simple balanced t (9;22). 9q34.1 region, (B) CytoCell MLL (KMT2A) Breakapart. The KMT2A (lysine methyltransferase 2A) gene at 11q23.3.

**Figure 2.**
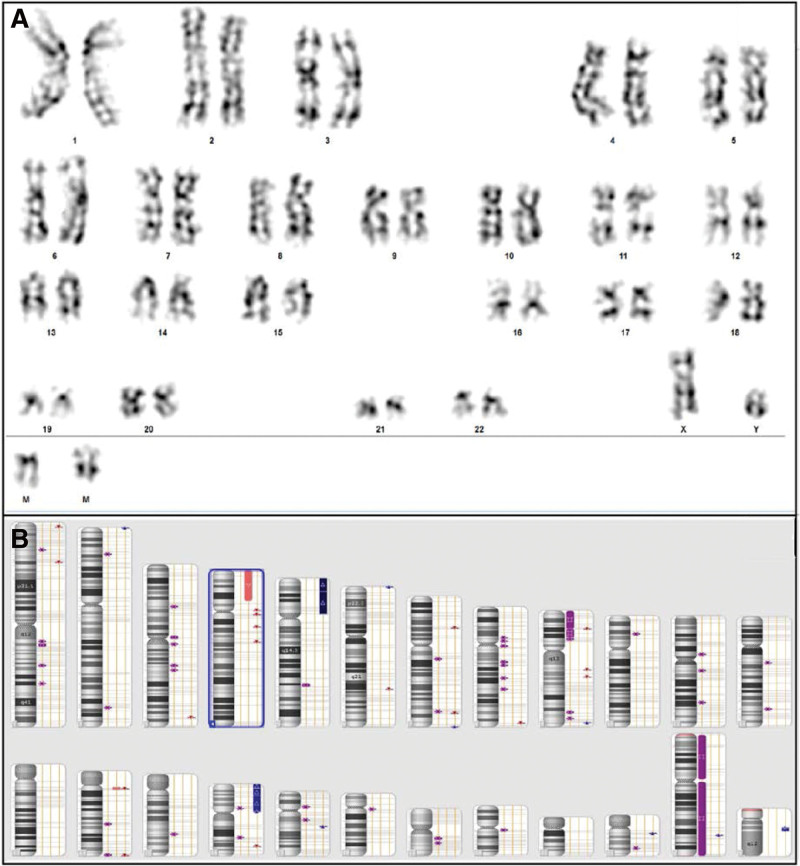
(A). Karyotype 48, XY, + mar1, + mar2 [16]/46, XY [4]. (B) The genetic microarray SNP analysis. (C) PRSS1 del biallelic. PRSS1 = serine protease 1.

**Figure 3.**
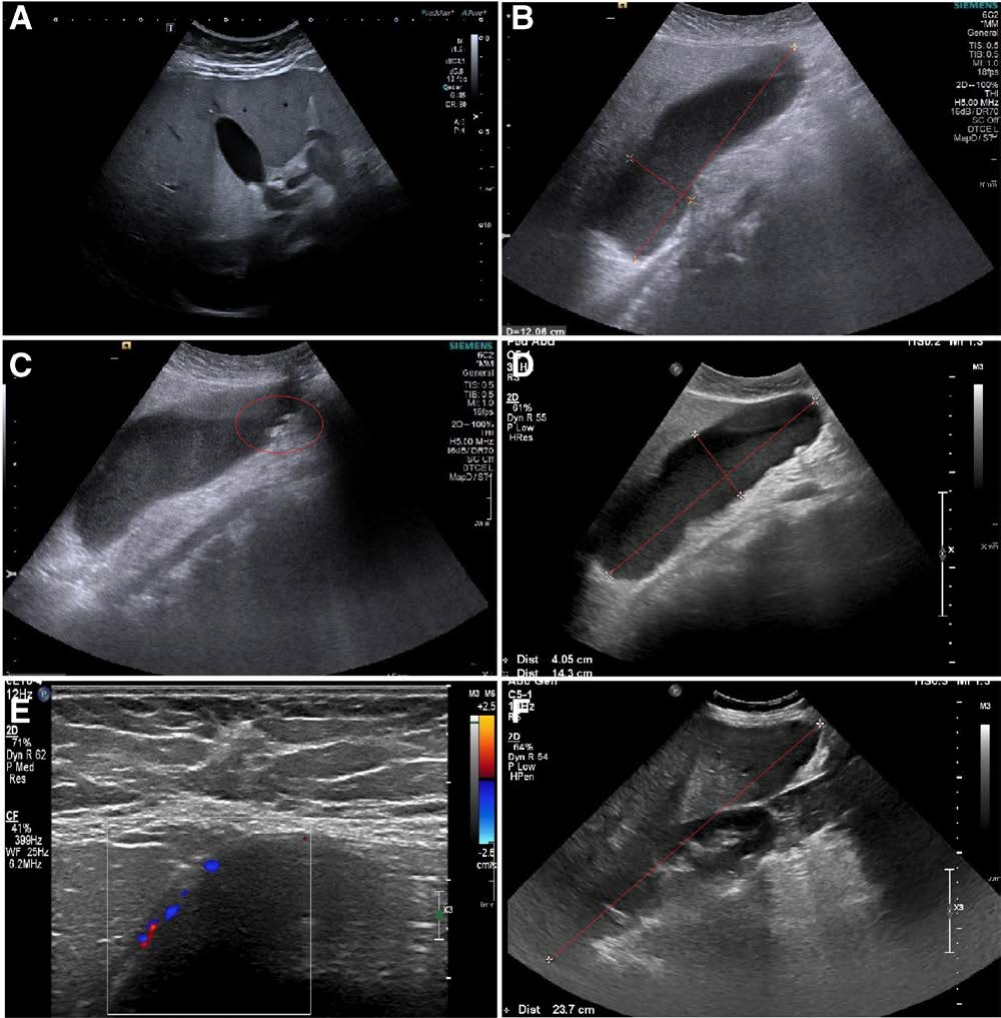
Abdominal ultrasound. (A) US of the abdominal cavity which had been done before treatment. Gallbladder without stones. The common bile duct and bile ducts were not dilated. (B) A gallbladder was measured approximately 121 × 47 mm (red tags) with a slightly echogenic bile. (C–D) The presence of several stones, up to 7 mm long (red tag). Common bile duct and bile ducts did not dilate. The gallbladder was reaching the level of the navel, of considerable size 146 × 52 mm (red tags), hydrocele lesion with the level of echogenic contents. Compression soreness in the projection of the gallbladder. (E) The gallbladder wall was marked, with slightly increased vascularization in Kolor Doppler. Compression soreness in the projection of the gallbladder. (F) A liver with a markedly elevated heterogeneous echogenicity, significantly enlarged, dim. in the front armpit line approx. 237 mm (red tag) (progression of dimensions). US = Ultrasound.

**Figure 4.**
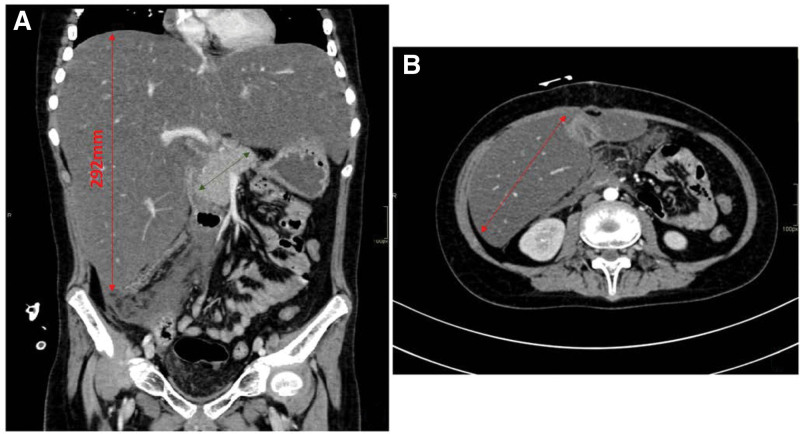
(A–B) Abdominal CT. Siemens Definition AS + spiral protocol before and after iv. admin-istration of a contrast agent. The liver was enlarged (dimension CC approx. 292 mm-red arrow), homogeneous, with clearly reduced density, without visible focal lesions. The gallbladder was partially constricted with signs of wall edema. In the lumen of the gallbladder there were 3 small gallstones 2–3.5 mm long. Pancreatic (green arrow) head was enlarged by dim. AP 29 mm with slightly blurred contours. The pancreatic parenchyma presented normal contrast enhancement (no areas of necrosis). Body and tail of pancreas were not enlarged, dim. AP 13 mm and 20 mm. Edema of adipose tissue and banded fluid areas were visible in the region of the head of the pancreas. In the area of the pancreas and along the mesentery of the intestine, there were moderately numerous lymph nodes with a diameter of in the short axis up to 10 mm. AP = acute pancreatitis, CT = computed tomography.

**Figure 5.**
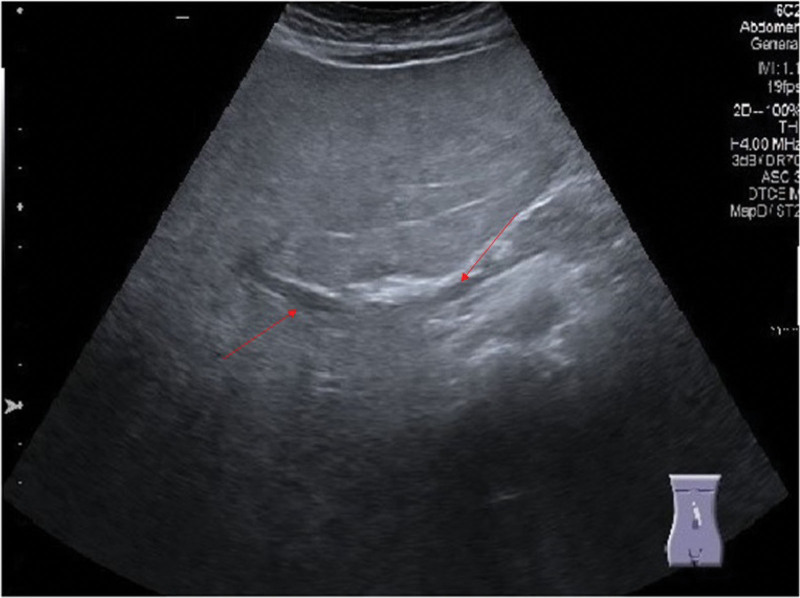
Abdominal ultrasound. Routine checkup. Status after cholecystectomy, surrounding area, liver parenchyma with lower echogenicity (red arrows). In addition, the liver was visible fragmentarily, enlarged, with increased echogenicity of the parenchyma. Intrahepatic bile ducts did not dilated. The pancreas in the area of the head was not enlarged, discreetly heterogeneous, otherwise obscured.

An 11-year-old boy presented with a 7-day history of flu-like illness. During ad-mission, the boy was in a generally severe condition, weakened, and apathetic. Physical examination revealed pale skin, with numerous ecchymoses on the skin of the whole body. He had no family history of chronic disease or cancers. The laboratory test results showed the following: white blood cell, 605 × 10^3/µL; neutrophile, 0.0 g/dL; hemoglobin level, 9.4 g/dL; platelet count, 56 × 10^3/µL; hemoglobin, 9.9g/dL; hematocrit, 29%; and 97% blastic cells. The immunophenotypes of lymphoid blasts were CD 45+, CD34+, CD7+, CD3+, TdT+, CD99+, and CD8+. The immunophenotype test indicated precursor T-cell acute lymphoblastic leukemia. The basic genetic tests, including fluorescence in situ hybridization, were performed: BCR: ABL1 (−) and KTM2A (−) (Fig. 1). Karyotype analysis showed a result of 48, XY, + mar1, + mar2 [16]/46, XY [4] (Fig. 2A). Additional genetic analysis microarray SNP indicated a complex karyotype: arr [GRCh37] 4p16.3p14 (68792_37010828) × 1 to 2, 5p15.33p12 (113576_43252270) × 4, 9p24.3p21..3 (192128_21818011) × 2 hmz, 9p21.3p13..3 (22017835_35870001) × 2 hmz, 16p13.3p11..1 (85880_34739571) × 3 (Fig. 2B). The patient had Biallelic mutation in the serine protease 1 (PRSS1) gene. Mutation in the PRSS1 is a genetic risk factor for pancreatitis.^[7]^ However, taking into consideration laboratory tests on cancer cells, it would be necessary to repeat the test after treatment in order to confirm mutation in cells. The most reliable test would be the 1 carried before the disease. In the preliminary abdominal ultrasound, a slightly enlarged liver was observed with a significant degree of steatosis, without focal changes in the dimensions of the anterior axillary line at 160 mm (Fig. 3A). The patient’s induction therapy was started with prednisone intravenous (60 mg/m2/day), daunorubicin per infusionem (p.i. 30 mg/m2), vincristine intravenous (1.5 mg/m2), polyethylene glycol (PEG)-asparaginase p.i. (2500 UI/m2), cyclophosphamide p.i. (1000 mg/m2), and methotrexate intra thecal (12 mg) according to the AIEOP-BFM ALL 2017 protocol IAp (International Collaborative Treatment Protocol for Children and Adolescents with Acute Lymphoblastic Leukemia). In the same period, due to increasing hyperleukocytosis (up to 725 10^3/µL), the patient was treated with leukapheresis for 2 days, with a good effect. During the treatment, fungal pneumonia was diagnosed, which caused a 5-day break in chemotherapy. Furthermore, chemotherapy was complicated by a deep bone marrow aplasia. On day 8 of therapy, there was 16% blastic cells in the patient’s peripheral blood, and on day 15, flow cytometry-minimal residual disease was 4.43 and 2.0% blastic cells in the bone marrow. On day 33 of treatment, polymerase chain reaction-minimal residual after induction was 2 × 10-4 and there was 0.0% blastic cells in yhr bone marrow. In the following days, on day 37 of therapy, the child had dyspnea, stomach pain and vomiting. Laboratory tests presented deep bone marrow aplasia, red blood cell count of 2.73 × 10^6/µL, a white blood cell count of 190 × 10^3/µL, a platelets count of 29 × 10^3/µL, hypokalemia of 2.62 meet/L, aspartate aminotransferase of 49 IU/L, alanine aminotransferase of 65 IU/L, hypertriglyceridemia of 1910 mg/dL, increasing gamma-glutamyl transpeptidase up to 1745 U/L, bilirubin of 3.01 mg/dL, hyperlipasemia of 500 U/L, hyperamylasemia of 206 U/L, procalcitonin of 10.66 ng/mL and C-Reactive Protein of 12.93 mg/L. In the ultrasound (US) test, the gallbladder measured approximately 121 × 47 mm with a slightly echogenic bile and the presence of several stones, up to 7 mm long (Fig. 3B). Cholecystitis and hepatitis were diagnosed. Broad-spectrum antibiotic therapy, potassium supplementation and hepatoprotective treatment (including L-ornithine L-aspartate) were administered. After 2 days, a controlled US test showed that lesions had progressed (Fig. 3C–F). Due to the patient’s serious condition, cholecystostomy was postponed, and initially, percutaneous drainage of the gallbladder was performed. After this procedure, an increase in the parameters of inflammation, amylase, lipase and a further increase in bilirubin and gamma-glutamyl transpeptidase were observed. On physical examination, severe pain was observed in the abdomen and limbs. Periodic vomiting of bile with blood was also observed. In addition, it was necessary to modify the patient’s broad-spectrum antibiotic therapy because of his poor response. Likewise, analgesic treatment and immunoglobulin infusions were administered. Moreover, there were further complications due to pancreatitis (Fig. 4A–B), and the boy was started on par-enteral nutrition. On account of gastro-esophageal bleeding, the boy received infusions of somatostatin and omeprazole. Because of severe bone marrow aplasia, the patient received granulocyte growth factor and a transfusion of blood products. In the following days, the boy’s general condition improved. Controlled abdominal ultrasound showed improvement in the general condition of the patient (Fig. 5). One month after the reported complaints, the patient underwent laparotomy cholecystectomy. The total interruption in chemotherapy was 74 days.

## 2. Discussion

Intensive chemotherapy causes different complications such as sepsis (46.50%), thrombosis (0.9–36.7%), bleeding (8.5%), neurological complications (3.6–11.0%), renal failure (9.1%), liver failure (1.1%) and pancreatitis (1.6–10.0%) which increase the length of the treatment protocol.^[2,6,8–11]^ Interruptions in treatment can have a negative effect on overall survival. During the induction phase, the delay in treatment in the first 8 days of the protocol (*P* < .01) was observed to have the greatest influence on outcome in a retrospective, single-center pediatric study with T-cell ALL patients treated following the ALLIC BFM 2002 and ALLIC BFM 2009 protocols. A total of 14 pediatric patients with T-ALL (82.35%) experience a delay during treatment, and out of these patients, 7 had a delay before the 8th day of treatment.^[12]^ In Mangum R. study, delays of 0 to 154 days during treatment were observed, with a median of 40 days. Extreme toxicities were noticed in 32 of 537 patients and were associated with worse relapse-free survival (2.9 vs 4.7 years; *P* < .001), and patients with T-ALL (*P* = .043) were at higher risk of developing side effects during the therapeutic protocol treatment. The study suggested that delays in treatment caused by intensive chemotherapy do not impact on survival, but do, however, contribute to many complications.^[13]^ In our patient we noticed a delay during the induction phase of ALL treatment, which can also be observed in the study presented below. In Agrawal V. study, a delay in the induction phase was observed in 70 (52.24%) patients, during consolidation in 78 (66.67%) patients, and during maintenance in 36 (42.35%) patients. The median follow-up was 41 months.^[14]^ In another study on a group of 141 pediatric patients, the median cumulative length of delay was 39 days, while the media delay in the intensive phase was 23 days and in the maintenance phase, 12 days.^[15]^ Interruption during chemotherapy was observed in 94.7% of patients.

Moreover, chemotherapy and myelosuppressive drugs are also risk factors for acute cholecystitis complications in cancer patients.^[16]^ Cholecystitis is a rare complication. In a cohort of pediatric ALL cases, cholecystitis was reported in 0.4% (n = 9) of patients, and 3 of them had gallstones.^[17]^ Abdominal pain, nausea, vomiting, diarrhea, and fever form a spectrum of symptoms of cholecystitis. In our study, a boy had calculous cholecystitis with several stones up to 7 mm long. In the literature, most pediatric cases describe the occurrence of acute acalculous cholecystitis during chemotherapy.^[17–19]^ However, in George J. single-center, retrospective study on a cohort of immunocompromised patients, 65.83% had acute calculous and US calculous lesions during diagnosis. In this study group, 80.80% (n = 97) of patients had leukemia or had received a recent bone marrow transplantation. The researcher suggests that most cases of cholecystitis in immunocompromised patients are calculous.^[20]^ Treatment for acute cholecystitis in ALL patients is dependent on the patient’s clinical status.^[20]^ Initially, our patient was prepared for laparoscopic cholecystectomy, but due to deep leucopenia and other complications such as pancreatitis and hepatitis, a laparotomy was performed. Percutaneous cholecystostomy is the treatment option of choice for critically ill patients with acute cholecystitis, while more stable patients may undergo definitive laparoscopic cholecystectomy.^[19–21]^ Xin et al^[22]^ presented a case study of an 11-year-old patient with B-ALL who developed intracranial hemorrhage and cholecystitis after completing high-dose methotrexate chemotherapy.

Acute pancreatitis (AP) is an established complication in ALL treatment, associated with the use of asparaginase.^[10]^ In Chen study, 4.00% (n = 353) of patients developed AAP (asparaginase-associated pancreatitis). High peak L-asparaginase dose (>45.000 U/m2/month) and diagnosis in older age (>6.80 years) were influences on AAP development.^[23]^ In Savage study, 1.60% of children hospitalized due to ALL developed pancreatitis. The risk of developing pancreatitis also increased with age.^[24]^ In the clinical diagnostic of AP, patients present the following: abdominal pain, serum amylase or lipase (3x the upper limit of normal) and/or imaging findings characteristic of AP. US for the initial imaging of AP was a limited diagnostic. Richardson study of clinically equivocal cases showed that a contrast-enhanced computed tomography or magnetic resonance imaging of the abdomen should be performed.^[25]^ The accurate mechanism of AAP is unknown. The genetic risk factors associated with asparaginase-associated pancreatitis in pediatrics that have been found are PRSS1, Unc-51 like autophagy activating kinase 2, regulator of g protein signaling 6, Carboxypeptidase A2, 4-hydroxy-2-oxoglutarate aldolase 1, metallopeptidase with thrombospondin type 1 motif 17, Myb-binding protein 1A, sperm antigen with calponin homology and coiled-coil domains 1, asparagine synthetase (glutamine-hydrolyzing) and cystic fibrosis transmembrane conductance regulator.^[26,27]^ Our patient had a mutation in the PRSS1 gene, encoding cationic trypsinogen (Fig.5). In the latest guidelines from the American College of Medical Genetics and Genomics, chronic pancreatitis (CP) was distinguished into a 5-category classification system (predisposing, likely predisposing, uncertain significance, likely benign and benign) and a 7 category classification system (pathogenic, likely pathogenic, predisposing, likely predisposing, uncertain significance, likely benign and benign). PRSS1, serine peptidase inhibitor Kazal type 1 and chymotrypsin C are specifically or highly expressed in the acinar cells of a patient with pancreatitis. Mutation in the PRSS1 gene results in CP-causing genes.^[28]^ Wolthers study showed the strongest association between trypsin-encoding serine protease 1 - serine protease 2 variations and the development of AAP. The minor allele of rs13228878 was previously found to reduce the risk of AAP.^[29]^ To confirm the mutation in the PRSS1 gene and the predisposition to CP, our patient had to undergo repeated genetic tests using somatic cells, not blast cells. An attempt has been made to develop procedures that would allow predicting AAP in advance using genetic variants. Nielsen used personalized artificial intelligence to predict very high risk and low risk of second AAP. They used germline single nucleotide polymorphisms from 1564 children with childhood ALL and an AAP case–control cohort (n = 205). The PRSS1/PRSS2 rs13228878 gene had the highest predictive features.^[27]^ Numerous complications in the boy in our case study caused a long break in the induction phase of chemotherapy, thus extending the duration of the main treatment. Currently, the patient remains in remission of hematological disease and is undergoing maintenance therapy.

AP and cholecystitis are rare but serious complications of chemotherapy. In the presented patient, severe sides effects caused a very long interruption (1.5 months) in the induction phase of chemotherapy. Complications during treatment require individual case analysis. Appropriate modification of treatment, dose reduction or discontinuation of medication should be taken into account. Abdominal US prior to the initiation of treatment allows for the subsequent assessment of changes over time, serving as a reference point. In the future, the assessment of genetic aberrations with an increased chance of developing complications in reaction to certain drugs will help to assess the risk during the treatment of ALL at an earlier stage of treatment.

## Author contributions

**Conceptualization:** Agata Rocka, Magdalena Woźniak, Monika Lejman, Joanna Zawitkowska.

**Writing – original draft:** Agata Rocka, Magdalena Woźniak, Monika Lejman, Joanna Zawitkowska.

**Writing – review & editing:** Agata Rocka, Magdalena Woźniak, Monika Lejman, Joanna Zawitkowska.
